# Introducing fuzziness into maximum common substructures for meaningful cluster characterisation

**DOI:** 10.1186/1758-2946-6-S1-P17

**Published:** 2014-03-11

**Authors:** Christian Herhaus

**Affiliations:** 1 Global Computational Chemistry, Merck Serono, Darmstadt, 64293, Germany

## 

Arranging similar structures in clusters is one of the typical tasks of modern Chemoinformatics with high impact in HTS follow-up, generation of structure activity relationships (SAR) and selection of starting points for compound optimisation. Methods for cluster generation are as diverse as the structures which they are applied to [1], may they be e.g. similarity- or substructure-based. Typically, medicinal chemists tend to orientate themselves in structure subsets like clusters with the help of substructures, so-called "scaffolds", which intuitively characterise the structural relationships between the molecules of the subset. In the case of substructure-based clustering, well established methods are existing for the generation of Maximum Common Substructures (MCS) which are present in all members of the structure population or a defined proportion thereof [2]. But in the case of similarity-based clusters, such MCS may either not be existing for the required dataset proportion or the common substructure may be so small that it is no longer representative and therefore meaningless.

The approach presented here allows the generation of MCS also for similarity-based clusters with a given inherent structural diversity. It does so by generating an MCS of reduced graphs in a first step, followed by mapping atom and bond indexes of this reduced MCS onto the full structures and aggregation of atom and bond information for each indexed atom/bond. In a final step, query features of the MDL SDF format (atom lists, query bonds) are utilized to map aggregated element and bond information onto the reduced MCS. As a result, "fuzziness" in atom and bond information is added to the MCS which, although still being fully database-searchable, is more meaningful for the characterisation of clusters as it can cover larger parts of the full structures than a conventional MCS could do. The approach was implemented in Pipeline Pilot™ for proof of concept but is general enough to be transferred to other technical platforms as well.
